# Supplementation with dietary omega-3 PUFA mitigates fetal brain inflammation and mitochondrial damage caused by high doses of sodium nitrite in maternal rats

**DOI:** 10.1371/journal.pone.0266084

**Published:** 2022-03-24

**Authors:** Jingchi Sun, Weishe Zhang

**Affiliations:** 1 Department of Obstetrics, Xiangya Hospital, Central South University, Changsha, Hunan, China; 2 Hunan Engineering Research Center of Early Life Development and Disease Prevention, Changsha, Hunan, China; Zagazig University, EGYPT

## Abstract

**Objective:**

Food safety and nutrition during pregnancy are important concerns related to fetal brain development. In the present study, we aimed to explore the effects of omega-3 polyunsaturated fatty acids (PUFA ω-3) on exogenous sodium nitrite intervention-induced fetal brain injury in pregnant rats.

**Methods:**

During pregnancy, rats were exposed to water containing sodium nitrite (0.05%, 0.15%, and 0.25%) to establish a fetal rat brain injury model. Inflammatory factors and oxidative stress levels were detected using enzyme-linked immunosorbent assay (ELISA) or flow cytometry. Subsequently, animals were divided into three groups: control, model, and 4% PUFA ω-3. Pregnancy outcomes were measured and recorded. Hematoxylin-eosin (H&E) staining and immunohistochemistry (IHC) were utilized to observe brain injury. ELISA, quantitative real-time PCR (qRT-PCR), western blot, flow cytometry, and transmission electron microscopy (TEM) were adopted to measure the levels of inflammatory factors, the NRF1/HMOX1 signaling pathway, and mitochondrial and oxidative stress damage.

**Results:**

With the increase of sodium nitrite concentration, the inflammatory factors and oxidative stress levels increased. Therefore, the high dose group was set as the model group for the following experiments. After PUFA ω-3 treatment, the fetal survival ratio, average body weight, and brain weight were elevated. The cells in the PUFA ω-3 group were more closely arranged and more round than the model. PUFA ω-3 treatment relieved inflammatory factors, oxidative stress levels, and mitochondria damage while increasing the indicators related to brain injury and NRF1/HMOX1 levels.

**Conclusions:**

Sodium nitrite exposure during pregnancy could cause brain damage in fetal rats. PUFA ω-3 might help alleviate brain inflammation, oxidative stress, and mitochondrial damage, possibly through the NRF1/HMOX1 signaling pathway. In conclusion, appropriately reducing sodium nitrite exposure and increasing PUFA omega-3 intake during pregnancy may benefit fetal brain development. These findings could further our understanding of nutrition and health during pregnancy.

## 1. Introduction

Food safety and nutrition during pregnancy remain important concerns, and maternal nutrition during pregnancy may have profoundly impact offspring development, especially brain development [1]. Poor nutrition, alcohol abuse, and other unsafe diets during pregnancy can impair brain development [2–4]. However, few in-depth studies have analyzed the impact of maternal nutrition on fetal brain development. In the present study, we examined the impact of exposure to an unsafe maternal diet during pregnancy on offspring’s brain development and attempted to reduce potential detrimental effects.

Nitrite occurs naturally in the food chain, and sodium and potassium nitrite are commonly approved food additives [5]. Individuals are most likely to experience unintentional exposure to nitrites. Nazarov et al. have demonstrated that nitrite can affect erythrocytes in the adult and umbilical cord blood [6]. Sodium nitrite is a potent oxidant that causes hypotension and restricts oxygen transport and delivery through methemoglobin formation, leading to hypoxia [7]. A previous study has found that excessive sodium nitrite exposure via drinking water or intraperitoneal injection could impair reproductive function and lead to congenital fetal disabilities [5]. In addition, exposure to sodium nitrite in drinking water has been associated with spontaneous abortion, growth restriction in utero, and various congenital disabilities [8]. Reportedly, mice administered 120 mg/kg/day sodium nitrite prior to pregnancy exhibited impaired reproductive ability [9]. Other studies have shown that exposure to 2 g/L of sodium nitrite during drinking water in the second half of pregnancy could impair the learning ability of offspring, which might be related to the neurotoxicity of sodium nitrite [10]. In the present study, we explored the effects of sodium nitrite on brain development in fetal rats.

Omega-3 polyunsaturated fatty acids (PUFA ω-3) is widely considered to affect neural development in fetuses and infants [11, 12]. For instance, maternal PUFA ω-3 supplementation can impact respiratory function in pups and fetal brain development [11, 13]. PUFA ω-3 accumulates in the brain before birth and during the neonatal period and is essential for the development and function of the central nervous system [14]. PUFA ω-3 is mainly composed of eicosapentaenoic acid (EPA), docosahexaenoic acid (DHA), and alpha-linolenic acid (ALA) [12]. Essential PUFA ω-3 reportedly regulates microglial phagocytosis of synaptic elements during animal brain development [15]. PUFA ω-3 is indispensable for brain development, as well as for preventing and treating behavioral, emotional, and other brain disorders [16]. Furthermore, a diet rich in PUFA ω-3 can provide beneficial anti-inflammatory effects [17]. Nevertheless, the effects of PUFA ω-3 on fetal brain dysplasia caused by sodium nitrite exposure during pregnancy have not been reported.

Herein, we first investigated the effects of exposure to different concentrations of sodium nitrite in pregnant rats by examining inflammation and oxidative stress in placental and cerebral cortex tissues of rat fetuses. Next, an appropriate concentration of sodium nitrite was used to construct a fetal rat brain injury model. PUFA w-3 was then added to the diet of pregnant rats. Subsequently, we examined whether PUFA ω-3 could alleviate exogenous sodium nitrite-induced fetal brain injury and possible underlying pathways.

## 2. Materials and methods

### 2.1. Experimental animals and treatment

We employed healthy adult Sprague Dawley (SD) rats (84 females and 84 males; weighing 190–210 g) in the present study. The rats had free access to food and water during the experimental period under a 12 h light/12 h dark cycle, housed in a special animal breeding environment. All animal experiments were approved by the Central South University Animal Welfare Ethics Committee (No. 202006068), and all methods were performed following the relevant guidelines and regulations. Male and female rats were mated in a 1:1 ratio, and vaginal emboli were observed to determine pregnancy. The appearance of a vaginal embolus was defined as the first embryonic day (E0). Male rats were separated after pregnancy, and pregnant female rats were housed in a single cage. Two protocols were designed for the experiment.

#### 2.1.1 Protocol 1: To select the appropriate concentration of sodium nitrite to construct a fetal injury model

Pregnant rats were randomly divided into four groups (12 rats/group). The rats in the control group drank normal water, whereas the other three groups of rats were provided low (0.05%, w/w), medium (0.15%, w/w), and high dose (0.25%, w/w) sodium nitrite water from E0 to E19, respectively [10, 18, 19]. Pregnant rats were anesthetized using isoflurane (2 mg/kg) [20]. Peripheral blood was collected from the veins of pregnant rats. Subsequently, the uterus and membranes were dissected, the placenta was separated from the fetus, and the umbilical cord was excised. The rats were euthanized by administering an intraperitoneal injection of pentobarbital (150 mg/kg). Fetuses and fetal brains were obtained. Blood samples were centrifuged at 3000 revolutions per minute (rpm) for 15 min to obtain serum and then stored at 4°C. An enzyme-linked immunosorbent assay (ELISA) was performed the following day. The cerebral cortex was immediately used for detecting reactive oxygen species (ROS). Based on these results, the group exhibiting the strongest oxidative stress effect was selected for subsequent experiments.

#### 2.1.2 Protocol 2: To investigate the effects of PUFA ω-3 on maternal sodium nitrite-exposed fetal rats

Pregnant rats were randomly divided into 3 groups (12 rats/group): control group, model group (high-dose, 0.25% sodium nitrite), and PUFA ω-3 group. The rats in the control group were provided normal drinking water and feed. The model group rats were administered high-concentration sodium nitrite water and normal feed. The PUFA ω-3 group rats were administered high-concentration sodium nitrite water and fed 4% PUFA ω-3 (Puritan’s Pride, USA, cat# 3832) [21, 22]. Sodium nitrite and PUFA ω-3 treatments were performed from E0 to E19. The experimental design is shown in S1 Fig.

Pregnant rats and fetuses were treated according to the above methods to harvest serum and tissues. The numbers of fetuses and live births were recorded. The sex of fetal rats was determined by measuring the distance between the anal and genital nodes; females presented approximately half the length of males. The fetuses and fetal brains of each group were weighed. The vital status of fetuses was distinguished based on their color and heartbeat; stillborn fetuses had no heartbeat and were reddish-brown in color. We selected the most vigorous fetus in each group for the follow-up study. Fetal brain sections were immobilized for pathological experiments, including hematoxylin-eosin (H&E) and immunohistochemistry (IHC), immunofluorescence (IF), and transmission electron microscopy (TEM) imaging. Portions of the cerebral cortex were immediately used for ROS and tetraethylbenzimidazole carbocyanine iodide (JC-1) detection and ELISA. Another portion was immobilized for IF and TEM measurements. The remaining portion of the fetal brain was maintained at -80°C for quantitative real-time PCR (qRT-PCR) and western blot.

### 2.2. ELISA

Placental tissue (approximately 70 mg) was treated with low-temperature ultrasound at 4°C in a 10% tissue homogenate and then centrifuged at 3000 rpm for 10 min to obtain the supernatant. Levels of tumor necrosis factor-alpha (TNF), inducible nitric oxide synthase (NOS2), interleukin-1beta (IL-1B), nitric oxide (NO), peroxynitrite (ONOO^-^), malondialdehyde (MDA), superoxide dismutase (SOD), adenosine triphosphate (ATP), mitochondrial complex II, and mitochondrial complex IV were detected using appropriate ELISA kits. Optical density (OD) was measured at 450 nm using an enzyme-plate analyzer (Shenzhen Huisongkeji, USA, cat# MB-530). Details of ELISA kits are listed in Table 1.

**Table 1:** Details of the ELISA kits.

Proteins	Manufacturers	Number of the Products	Place of origin
TNF	CUSABIO BIOTECH	CSB-E11987r	China
NOS2	CUSABIO BIOTECH	CSB-E08325r	China
IL-1B	CUSABIO BIOTECH	CSB-E08055r	China
NO	NanJing JianCheng	A013-2	China
ONOO^-^	Jianglaibio	JL21035	China
MDA	Jianglaibio	A003-1-1	China
SOD	Jianglaibio	A001-3-1	China
ATP	NanJing JianCheng	A095-1-1	China
mitochondrial complex Ⅱ	Suzhou Comin	FHTB-1-γ	China
mitochondrial complex Ⅳ	Suzhou Comin	FHTD-1-γ	China

### 2.3. ROS analysis

The fetal brain cerebral cortex was cut, crushed, and digested with 4 mL of 0.25% trypsin (containing 0.02% ethylene diamine tetraacetic acid) at 37°C for 1 h. A Pasteur pipette was used to blow away tissue and disperse cells. After filtration through a 70-μm cell filter, the filtrate was collected and centrifuged at 1,500 rpm for 5 min to collect the cell precipitate. The cells were washed with Roswell Park Memorial Institute 1640 complete medium, centrifuged at 1,500 rpm for 5 min, and the supernatant was discarded. Diluted DCFH-DA (Beyotime Biotechnology, China, cat# S0033) in serum-free medium (1:1000) was added to the cell precipitate to resuspend cells, followed by incubation at 37°C for 20 min in the dark. The cells were collected and washed in a serum-free medium. Approximately 10,000 cells were collected for flow cytometric analysis. The green fluorescence of ROS-FITC (excitation wavelength Ex = 488 nm, emission wavelength Em = 530 nm) was detected by flow cytometry (Beckman, USA, cat# A00-1-1102) using the FITC channel.

### 2.4. H&E staining

The fetal brain was fixed, paraffin-embedded, and cut into 4-μm slices. Brain sections were stained with H&E and observed under a microscope (Cat # BA210T, Motic, China) [23]. Representative images were obtained at low and high magnifications (100× and 400×, respectively).

### 2.5. Immunohistochemistry

The fetal brain was fixed, paraffin-embedded, and cut into 4-μm slices. After thermal antigen repair, the sections were incubated with primary antibodies against OCLN (1:200; Proteintech, USA, cat #27260-1-AP) and tight junction protein 1 (TJP1) (1:200; Proteintech, USA, cat #21773-1-AP) overnight at 4°C. After washing with phosphate-buffered saline (PBS), samples were incubated with a secondary antibody (100 μL; ZSGB-BIO, China, cat # PV-9001). Representative images were obtained under a microscope (Cat # BA210T, Motic, China) at low and high magnification (100× and 400×, respectively). Image-Pro Plus software was used to analyze the images.

### 2.6. qRT-PCR

Cerebral cortices (approximately 0.025 g) of fetal rats from different treatment groups were collected. Total RNA (approximately 20 μg) was extracted with TRIzol reagent (cat #15596026, Thermo Fisher Scientific), and cDNA was synthesized using the Hifiscript cDNA Synthesis Kit (Cwbiotech, China, cat# CW2569). Primers for *Tnf*, *Nos2*, *Il1b*, *nuclear respiratory factor 1 (Nrf1)*, *heme oxygenase 1* (*Hmox1)*, *Actb*, *cytochrome c oxidase I*, *mitochondrial (mt-Co1)*, *and succinate dehydrogenase complex flavoprotein subunit A (Sdha)* were designed using the Primer5 software after searching for target gene mRNA sequences in NCBI. *Sdha* and *Actb* were used as internal controls, and relative mRNA expression levels were analyzed using the 2^−ΔΔCT^ method. Primer sequences used for qRT-PCR are listed in Table 2.

**Table 2:** Primer sequences used for qRT-PCR.

Gene	Sequences (5’-3’)	Accession number	Product length (bp)
*Tnf*	F:CCCCTCTATTTATAATTGCACCT	24835	167
	R:CTGGTAGTTTAGCTCCGTTT		
*Il1b*	F:TGTGATGTTCCCATTAGAC	24494	131
	R:AATACCACTTGTTGGCTTA		
*Nos2*	F:TTCAGCTACGCCTTCAACACC	24599	109
	R:CTCCATTGCCAAATGTGCTTG		
*Nrf1*	F:TACAAGGCGGGGGACAGATA	312195	101
	R:TGCATGAACTCCATCTGGGC		
*Hmox1*	F:TTGTTATTTCCCCAGTTCTACCAG	24451	87
	R:CAAAAGACAGCCCTACTTGGTT		
*Actb*	F:ACATCCGTAAAGACCTCTATGCC	11461	223
	R:TACTCCTGCTTGCTGATCCAC		
*mt-Co1*	F:ACATCTTAATTCTTCCAGGGTTTG	26195	190
	R:GATGTAAAGTAGGCTCGGGTGT		
*Sdha*	F:TGACCTTAGCCAGTCCAGTCTC		
	R: CACTACTGTCCAAGGGATTGCTA	157074	184

### 2.7. Western blot

Fetal cerebral cortex tissues (approximately 0.025 g) from different treatment groups were collected. Protein samples (20 μg) were separated by 12% sodium dodecyl sulfate-polyacrylamide gel electrophoresis (SDS-PAGE). The isolated protein was transferred to a polyvinylidene fluoride membrane activated with methanol, blocked with 5% skim milk, dried at room temperature for at least 1 h, and incubated with the primary antibody overnight at 4°C. Primary antibodies used for incubation were anti-TNF, anti-IL-1B, anti-NOS2, anti-NRF1, anti-HMOX1, and anti-ACTB. The sections were then incubated with secondary anti-IgG antibodies at 37°C for 90 min. Chemiluminescence (Millipore, USA) was visualized and analyzed using imaging software (GE Healthcare Life Sciences, USA). Details of antibodies against the proteins are shown in Table 3.

**Table 3:** Details of the antibodies against the proteins in western blot.

Antibodies	Manufacturers	Number of the products	Place of origin	Dilutions
TNF	Proteintech	17590-1-AP	USA	1:500
NOS2	Proteintech	18985-1-AP	USA	1:500
IL-1B	Proteintech	16806-1-AP	USA	1:500
NRF1	Proteintech	66832-1-Ig	USA	1:10000
HMOX1	Proteintech	27282-1-AP	USA	1:1500
ACTB	Proteintech	66009-1-Ig	USA	1:5000
IgG	ProteinTech	SA00001-1 or SA00001-2	USA	1:5000 or 1:6000

### 2.8. IF

The cerebral cortices of the fetal rats were collected for paraffin sectioning. The sections were then deparaffinized, followed by antigen repair, and placed in sodium borohydride solution for 30 min at room temperature. Subsequently, sections were placed in Sudan black dye solution at room temperature for 5 min and rinsed with water. Processed tissues were blocked at 37°C in 5% bovine serum albumin for 60 min and incubated overnight with the primary antibody at 4°C. The primary antibodies used for incubation included anti-sex determining region Y-box 2 (SOX2; 1:50), anti-enolase 2 (ENO2; 1:50), and anti-tubulin beta 3 class III (TUBB3; 1:50). The tissues were then incubated with a secondary anti-IgG antibody (1:500) at 37°C for 90 min and rinsed with PBS. Nuclei were stained with DAPI working solution at 37°C for 10 min and rinsed with PBS. The slides were mounted in buffered glycerol and observed under a fluorescence microscope (Cat # BA410T; Motic, China). Details of antibodies against the proteins are shown in Table 4.

**Table 4:** Details of antibodies against the proteins in IF.

Antibodies	Manufacturers	Number of the Products	Place of origin	Dilutions
SOX2	Abcam	ab93689	USA	1:50
ENO2	ProteinTech	66150-1-Ig	USA	1:50
TUBB3	Abcam	ab78078	USA	1:50
IgG	ProteinTech	SA00013-3 or SA00013-4	USA	1:500

### 2.9. JC-1 analysis

JC-1 was diluted (Beyotime Biotechnology, China; cat# C2006) according to the manufacturer’s instructions. Then, 2 mL of JC-1 staining buffer (5×) was added and mixed to obtain JC-1 working fluid. To obtain JC-1 staining buffer (1×), 4 mL ultrapure water was added to 1 mL of JC-1 staining buffer (5×). Resuspended cells were obtained from cortical tissues using the treatment described above, and JC-1 staining solution (0.5 mL) was added, reversed, and mixed several times. The cells were incubated at 37°C for 20 min and centrifuged at 1,000 rpm for 4 min. Subsequently, cells were washed with JC-1 staining buffer (1×) and resuspended. On detecting the JC-1 monomer, the excitation light was set at 490 nm, while the emission light was set at 530 nm for flow cytometry detection. Flow cytometry (Beckman, USA, cat. # A00-1-1102) was performed for JC-1 polymers by setting the excitation and emission wavelengths at 525 and 590 nm, respectively.

### 2.10. TEM

As previously described, one cubic centimeter of cortical tissue was fixed with 4% glutaraldehyde, followed by dehydration, embedding, and ultrathin sectioning [24]. The tissue ultrastructure was observed using TEM (Hitachi, Japan, H-7700).

### 2.11. Statistical analysis

Statistical analyses were performed using GraphPad Prism 8.0 (GraphPad Software, Inc., La Jolla, CA, USA). One-way analysis of variance (ANOVA) was used to determine statistical significance between experimental groups, followed by a post-hoc Tukey’s test. All data are presented as mean ± standard deviation. Statistical significance was set at *P* < 0.05. All experiments were performed in triplicate.

## 3. Results

### 3.1. Exposure to sodium nitrite during pregnancy induced inflammation and oxidative stress

**Fig 1:**
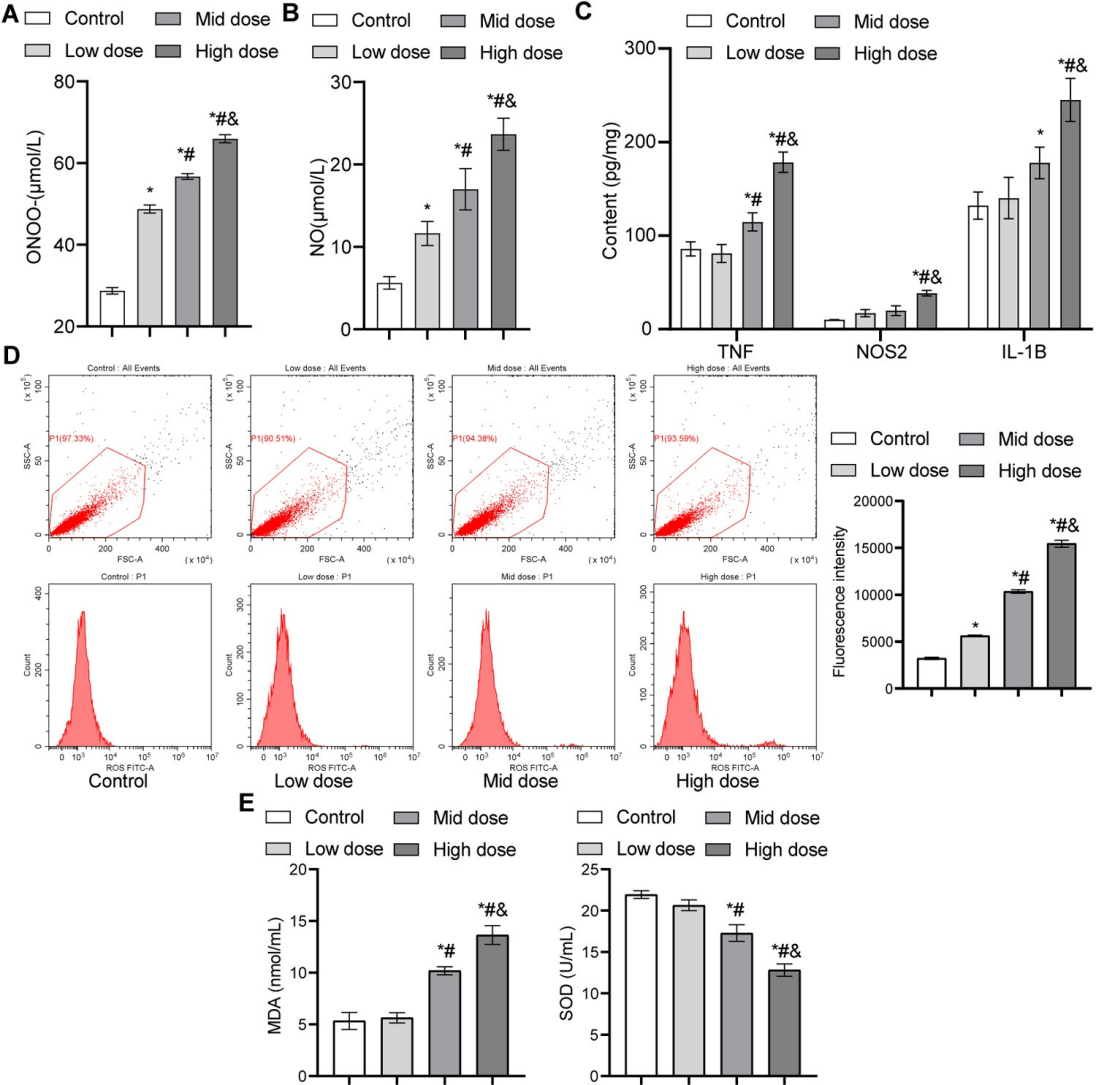
Exposure to sodium nitrite during pregnancy induces inflammation and oxidative stress. (A) The concentration of plasma peroxynitrite in pregnant rats as detected by ELISA, n = 12 rats/group. (B) Plasma NO levels in pregnant rats from different groups were detected by ELISA, n = 12 rats/group. (C) Effect of sodium nitrite exposure on blood inflammation in placenta tissues as detected by ELISA, n = 3 rats/group. (D) Flow cytometry was used to detect ROS in the fetal cerebral cortex, n = 3 rats/group. (E) Plasma MDA and SOD levels of fetal rats in different groups as detected by ELISA, n = 3 rats/group. *P < 0.05 *vs*. control group; ^#^*P* < 0.05 *vs*. low dose group; ^&^*P* < 0.05 *vs*. medium-dose group. MDA, malondialdehyde; NO, nitric oxide; ROS, reactive oxygen species; SOD, superoxide dismutase.

Pregnant rats were fed different concentrations of sodium nitrite, and peripheral blood was collected to determine the NOO^-^ and NO concentrations. Compared with the control group, NOO^-^ and NO concentrations in the low, mid, and high dose groups were significantly increased in a dose-dependent manner (**Fig 1A and 1B**). We then explored the effects of sodium nitrite on placental inflammation by assessing TNF, NOS2, and IL-1B as indicators of inflammation [25]. The concentrations of TNF, NOS2, and IL-1B were higher in the low, mid, and high dose groups than those in the control group, and the effect was dose-dependent. Compared with the low and mid dose groups, the high dose group exhibited significantly higher TNF, NOS2, and IL-1B levels (**Fig 1C**). We examined the effects of sodium nitrite-induced oxidative stress on fetal cerebral cortex tissue. Compared with the control group, the levels of ROS and MDA showed an upward trend in the sodium nitrite-treated group and were markedly increased in the high dose group (**Fig 1D and 1E**). However, the SOD concentration showed a downward trend following sodium nitrite treatment, with the most pronounced effect observed in the high dose group (**Fig 1E**). These results suggested that excessive sodium nitrite exposure leads to oxidative stress, injury, and inflammation. In addition, the high dose group presented the most serious oxidative stress damage to the brains of fetal rats; therefore, we selected the high dose group as the model group for the following experiments.

### 3.2. PUFA ω-3 ameliorated adverse pregnancy outcomes following sodium nitrite exposure during pregnancy

**Fig 2:**
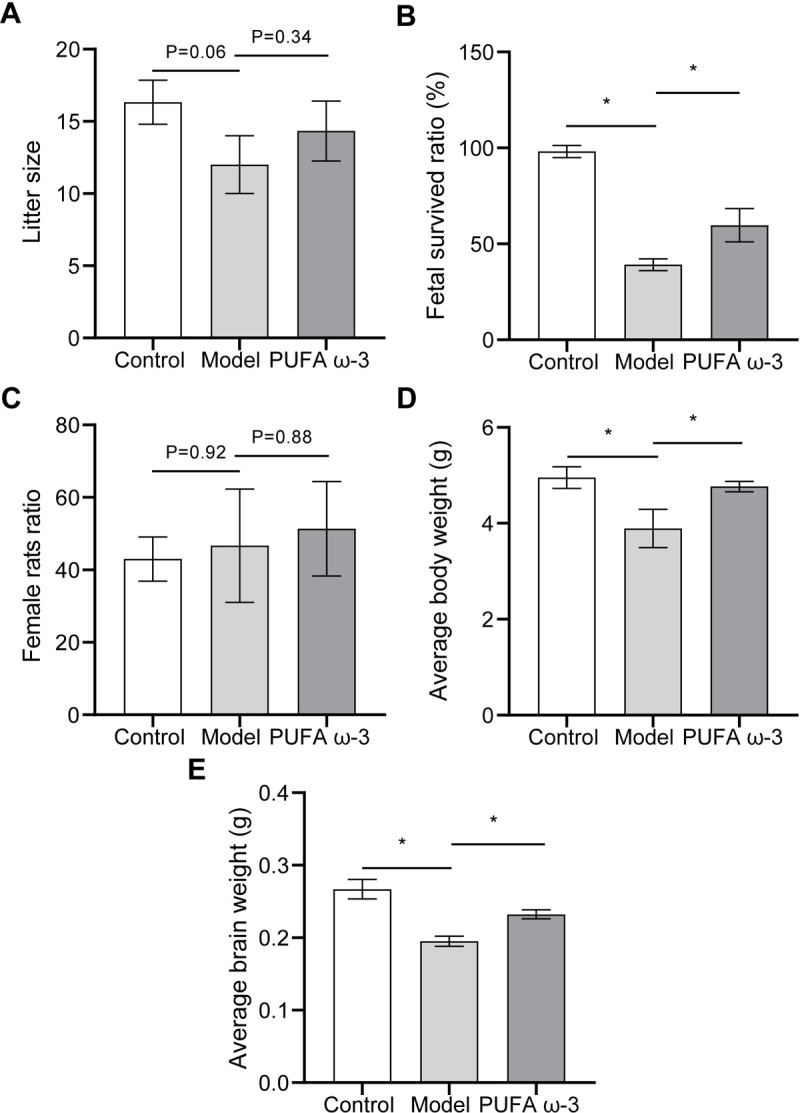
PUFA ω-3 ameliorates adverse pregnancy outcomes following sodium nitrite exposure during pregnancy. (A) The number of fetuses per litter, (B) The fetal survived ratio, (C) female rats ratio, (D) average body weight, and (E) average brain weight were measured and recorded. *P < 0.05, n = 3 rats/group.

We further examined the therapeutic effects of PUFA ω-3 on sodium nitrite-induced injury in pregnant rats. Accordingly, the number of fetuses per litter, the number of live fetuses, sex, weight, and brain weight were measured and recorded. PUFA ω-3 treatment alleviated the downward trend in litter size induced by sodium nitrite (**Fig 2A**). The fetal survived ratio was lower in the model group than in the control group; however, this ratio was higher in the PUFA ω-3 group than in the model group **(Fig 2B)**. We observed no significant difference in the ratio of female rats (**Fig 2C**). Based on the observed results, the average fetal body and brain weights were significantly decreased after sodium nitrite exposure. Compared with the model group, average fetal body and brain weights were significantly increased in the PUFA ω-3 group (**Fig 2D and 2E**). These findings suggested that ω-3 ameliorated adverse pregnancy outcomes following sodium nitrite exposure during pregnancy.

### 3.3. PUFA ω-3 alleviated fetal brain damage induced by sodium nitrite exposure during pregnancy

**Fig 3:**
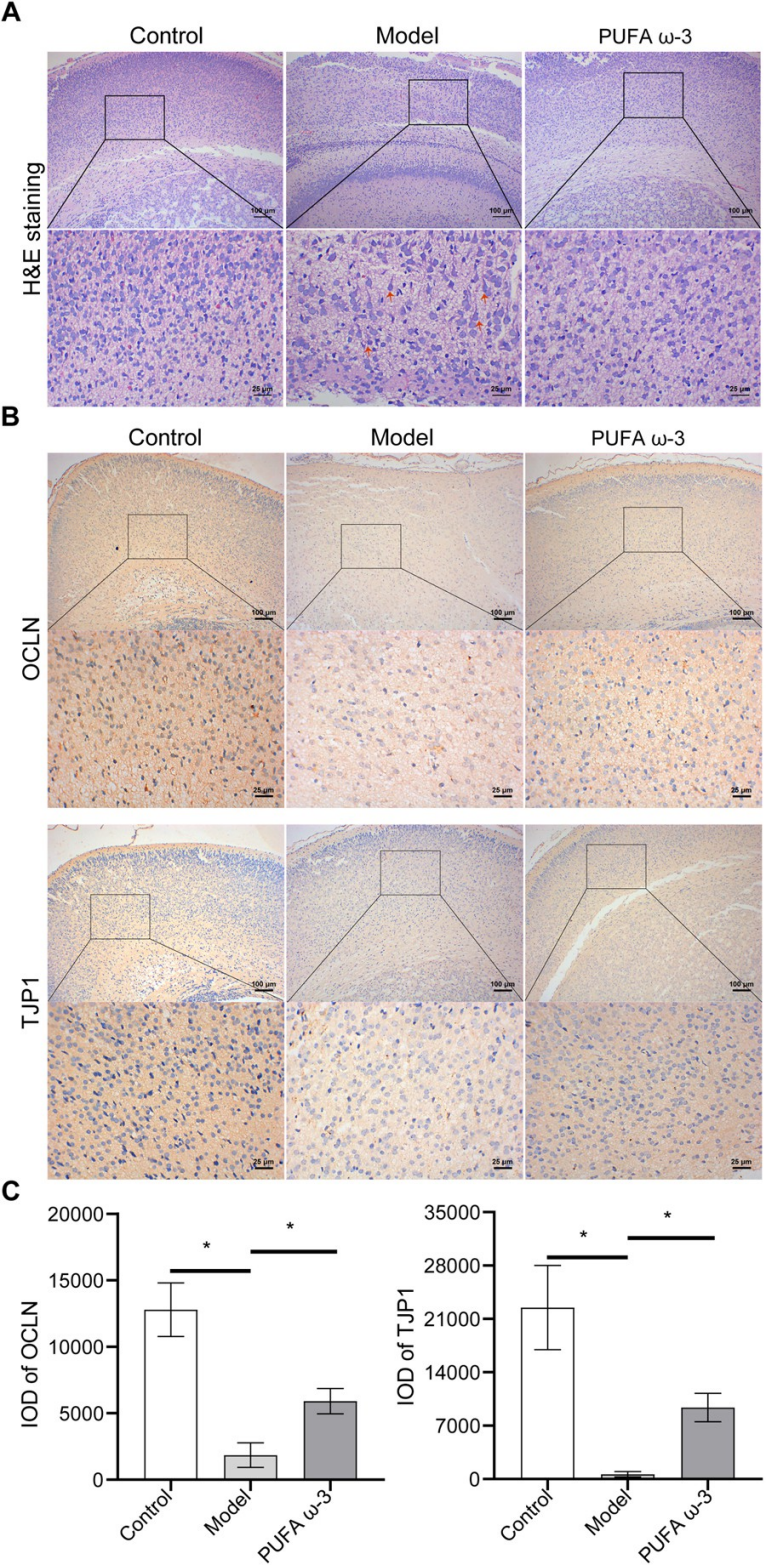
PUFA ω-3 alleviates fetal brain damage induced by sodium nitrite exposure during pregnancy. (A) Representative images of brain tissues from each group were analyzed by H&E staining, n = 3 rats/group. (B) Representative images of OCLN and TJP1 expression in brain tissues of each group, as measured by immunohistochemistry, n = 3 rats/group. Blue indicates nuclei. Light red indicates cytoplasm. Red arrows indicate typical nuclear damage. (C) Integrated optical density (IOD) values of representative images of OCLN and TJP1 expression in brain tissues of each group were analyzed by Image-Pro-Plus, n = 3 rats/group. *P < 0.05. H&E, hematoxylin-eosin.

We further investigated the effects of PUFA ω-3 on brain tissue injury caused by sodium nitrite. Based on H&E staining, the control group presented closely arranged, numerous cells with round nuclei. In the model group, cells were loosely organized, the intercellular space was increased, the number of cells decreased, and some cells became longer and tailed, indicating the damaging effects of sodium nitrite on cerebral cortex cells. Compared with the model group, the PUFA ω-3 group displayed more closely arranged and numerous cells (**Fig 3A**). To further investigate the effects of PUFA ω-3, tight junction proteins (OCLN and TJP1) were detected by IHC. The model group had significantly lower OCLN and TJP1 levels than the control group. In contrast, the PUFA ω-3 group had substantially higher OCLN and TJP1 levels than the model group (**Fig 3B and 3C**). These results indicated that PUFA ω-3 alleviates fetal brain damage and impairs the blood-brain barrier (BBB) function induced by sodium nitrite exposure during pregnancy.

### 3.4. PUFA ω-3 alleviated cerebral cortical tissue damage induced by sodium nitrite exposure via the NRF1/HMOX1 pathway in fetal rats

**Fig 4:**
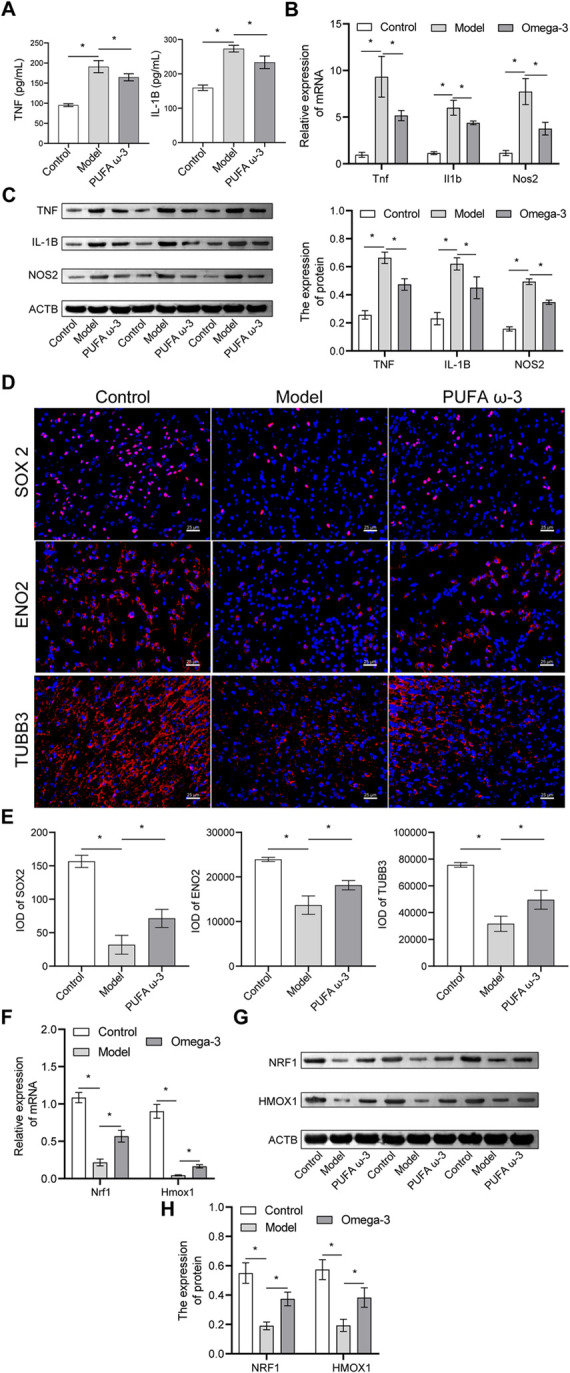
PUFA ω-3 alleviates sodium nitrite exposure-induced cerebral cortical tissue damage in fetal rats via the NRF1/HMOX1 pathway. (A) TNF and IL-1B concentrations were detected by ELISA, n = 3 rats/group. (B-C) The mRNA and protein expression levels of *Tnf*, *Nos2*, *and Il1b* were detected by qRT-PCR and western blot, n = 3 rats/group. Original blots are presented in S2A Fig. (D) Expression levels of SOX2, ENO2, and TUBB3 in the brain cortical tissue of each group were detected by IF, n = 3 rats/group. (E) IOD data statistics of SOX2, ENO2, and TUBB3 expression in the brain cortical tissue of each group were analyzed by Image-Pro-Plus, n = 3 rats/group. (F-H) *Nrf1 and Hmox1* mRNA and protein expression levels were analyzed by qRT-PCR and western blot, n = 3 rats/group. Original blots are presented in S2B Fig. *P < 0.05. IF, immunofluorescence; IL-1B, interleukin-1 beta; IOD, integrated optical density; qRT-PCR, quantitative real-time PCR; TNF, tumor necrosis factor.

To further investigate the effects of PUFA ω-3 on fetal inflammation, inflammatory factors were examined in placental and cerebral cortex tissues. Exposure to sodium nitrite increased TNF and IL-1B levels in placental tissues. TNF and IL-1B concentrations were lower in the PUFA ω-3 group than in the model group (**Fig 4A**). PUFA ω-3 alleviated the increased mRNA and protein expression levels of *Tnf*, *Nos2*, *and Il1b* in cerebral cortex tissues (**Fig 4B and 4C**). The transcription factor SOX2 plays an important role during the early development of mammalian organs [26]. The expression of SOX2, ENO2, and TUBB3 in the cerebral cortex of fetal rats was analyzed using IF. Compared with the model group, SOX2, ENO2, and TUBB3 expression increased significantly in the PUFA ω-3 group **(Fig 4D and 4E)**. To further explore the therapeutic mechanism of PUFA ω-3, we measured the NRF1/HMOX1 protein expression, a classical pathway associated with oxidative stress [27, 28]. PUFA ω-3 upregulated *Nrf1 and Hmox1* expression when compared with that in the model group **(Fig 4F and 4G)**. These results indicated that PUFA ω-3 might alleviate sodium nitrite exposure-induced cerebral cortical tissue damage in fetal rats via the NRF1/HMOX1 pathway.

### 3.5. PUFA ω-3 mitigated mitochondrial damage in fetal cortical tissue induced by sodium nitrite exposure during pregnancy

**Fig 5:**
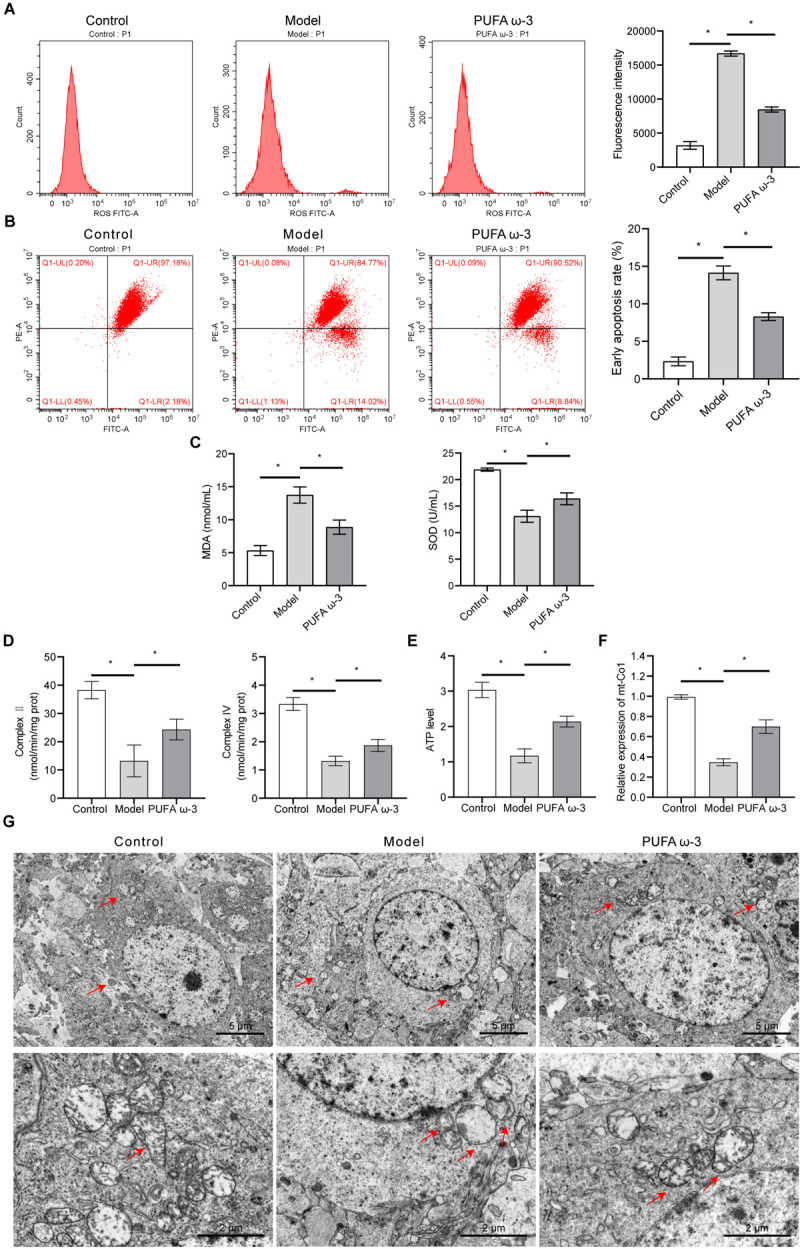
PUFA ω-3 reduces mitochondrial damage induced by sodium nitrite exposure in fetal cortical tissue. (A-B) ROS and JC-1 levels in the cerebral cortex of fetal rats as determined by flow cytometry, n = 3 rats/group. (C) Plasma MDA and SOD levels of fetal rats in different groups were detected by ELISA, n = 3 rats/group. (D-E) Mitochondrial complex II, mitochondrial complex IV, and ATP activity were measured by ELISA, n = 3 rats/group. (F) *mt-Co1* expression detected by qRT-PCR, n = 3 rats/group. (G) Mitochondrial damage observed using TEM, n = 3 rats/group. Red arrows indicate mitochondria. Scale bars: 2 μm and 5 μm. *P < 0.05. MDA, malondialdehyde; qRT-PCR, quantitative real-time PCR; ROS, reactive oxygen species; SOD, superoxide dismutase; TEM, transmission electron microscopy.

**Fig 6:**
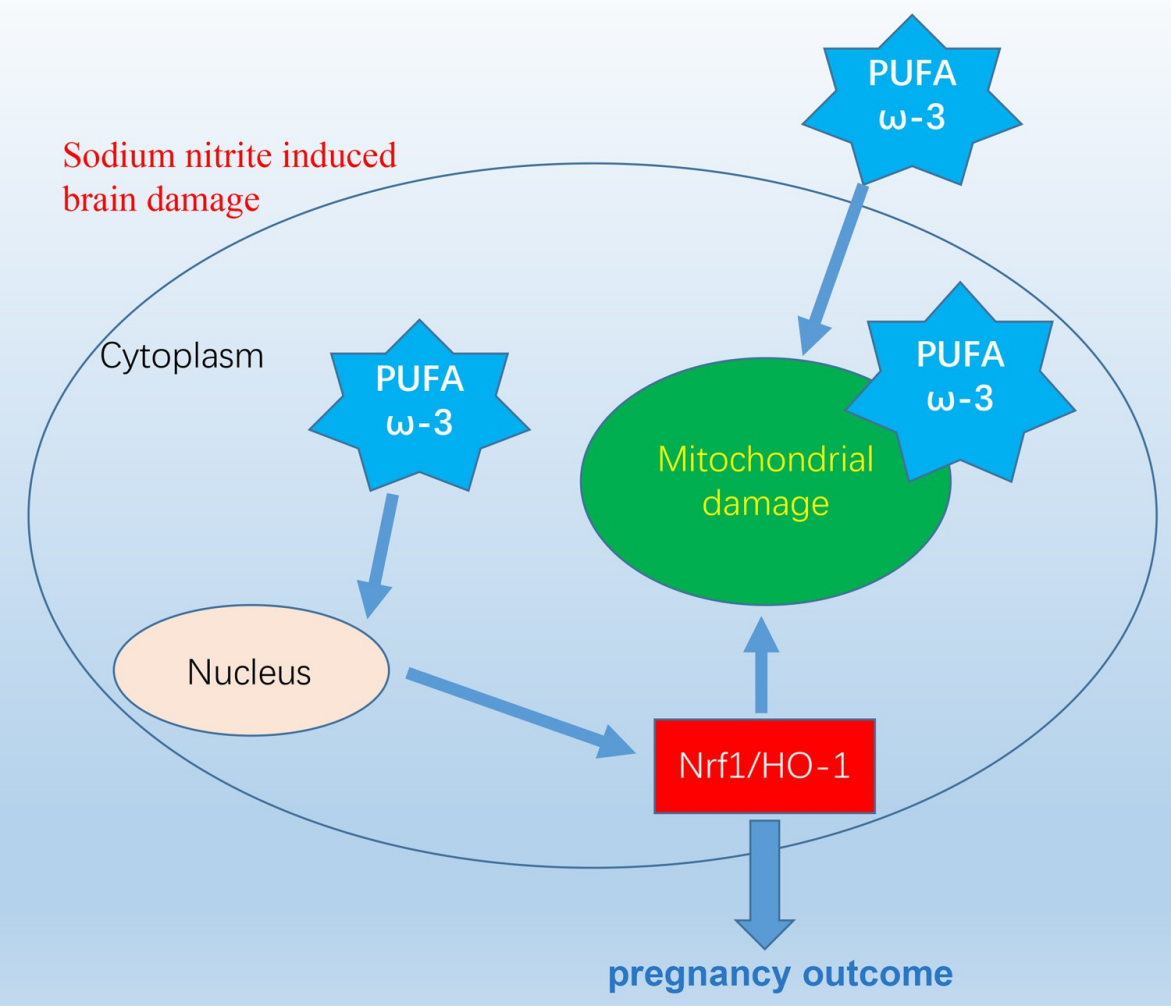
PUFA ω-3 may alleviate brain damage in fetal rats caused by sodium nitrite exposure during pregnancy via NRF1/HMOX1 signaling.

Mitochondrial function was assessed using flow cytometry. Exposure to sodium nitrite increased ROS and JC-1 levels in fetal cortical tissues; these levels were decreased after PUFA ω-3 supplementation **(Fig 5A and 5B)**. PUFA ω-3 mitigated the increased MDA concentration and decreased SOD concentration induced by sodium nitrite **(Fig 5C)**. Compared with the control group, the model group demonstrated reduced complex II and IV activities. Compared with the model group, complex II, complex IV, and ATP activities were significantly elevated in the PUFA ω-3 group **(Fig 5D and 5E)**. qRT-PCR was used to analyze the mitochondrial *mt-Co1* gene expression, and the results revealed that sodium nitrite exposure decreased *mt-Co1* mRNA expression. *The mt-Co1* mRNA expression was significantly higher in the PUFA ω-3 group than in the model group **(Fig 5F)**. TEM was used to determine the effect of PUFA ω-3 on the mitochondrial oxidative stress damage caused by sodium nitrite. Compared with the control group, the model group showed a reduced number of mitochondria and damage to the internal structure of the mitochondria. Compared with the control group, the model group displayed a blurred mitochondrial membrane, along with a severely damaged mitochondrial structure. Notably, mitochondrial damage was mitigated in the PUFA ω-3 group **(Fig 5G)**. The results illustrated that PUFA ω-3 could reduce the mitochondrial damage induced by sodium nitrite exposure in fetal cortical tissue. **Fig 6** presents the possible underlying mechanism through which PUFA ω-3 alleviates sodium nitrite-induced damage.

## 4. Discussion

During pregnancy and lactation, the administration of sodium nitrite in drinking water can seriously affect erythropoiesis, growth, and mortality of the progeny while also endangering organ development and neurological function [29]. In inflamed tissues, the reaction between NO and superoxide forms ONOO^-^ [30], leading to cell signal transduction, oxidative stress injury, and cell necrosis or apoptosis [31]. In the present study, sodium nitrite exposure increased NOO^-^, NO, TNF-, NOS2, and IL-1B. ROS and MDA levels increased, while SOD levels decreased in the fetus, resulting in oxidative stress. These findings indicate that overexposure to sodium nitrite during pregnancy induces inflammation and oxidative stress.

In previous studies, rats were administered sodium nitrite from E13 to E21 [10]. Pregnant rats were reportedly exposed to low-level heavy metal mixtures in drinking water throughout pregnancy and lactation, causing cognitive deficits and impairments in respective offspring [32, 33]. In one report, pregnant rats were exposed to drinking water containing 1% lead acetate from the first day of pregnancy to the weaning of offspring. The authors found that this treatment protocol caused synaptic dysfunction in the offspring, further inducing neurotransmission abnormalities and neuronal dysfunction [34]. In the present study, we set the sodium nitrite concentrations as 0.05, 0.15, and 0.25% by performing preliminary experiments. Similar to previous studies, the exposure time ranged from E0-E19. The designed sodium nitrite exposure protocol employed in the present study affords the following advantages. (ⅰ) Compared with treatment at a certain time during pregnancy, sodium nitrite exposure during pregnancy was more in line with the real-world situation and can simulate the toxicological effects in the human environment, which was more realistic; (ⅱ) the setting of different concentration gradients of sodium nitrite was more conducive to show the effects of sodium nitrite on the nervous system of fetal rats; (ⅲ) the exposure dose range designed in the present study was wider based on previous studies, which was more conducive to discover possible induced damage. Given time constraints, we did not examine the effects of sodium nitrite on fetal rats at other pregnancy time points. Additional studies are warranted to further investigate the effects of sodium nitrite exposure during different gestational periods in fetal rats.

The brain is well-known to control sensation, cognition, memory, and motor functions. The production of cytotoxic factors (such as ONOO^-^) can induce the death of adjacent dopaminergic neurons and mitochondrial dysfunction, further damaging brain functions [35]. Herein, sodium nitrite stimulation led to a loose arrangement of cerebral cortical cells, enlarged intercellular spaces, decreased cell number, few longer and tailed cells, damaged mitochondrial structure, and reduced OCLN and TJP1 levels, suggesting impairment of fetal rat brain cortical tissue, mitochondria, and BBB function. In addition, the fetal rat brain exhibited oxidative stress and inflammation, along with altered levels of mitochondria-related enzymes and neuronal injury indicators. Conversely, omega-3 PUFAs mitigated these effects of sodium nitrite. In future studies, we plan to investigate the effects of sodium nitrite and PUFA ω-3 on fetal rat brain functions.

PUFA ω-3 reportedly possesses the following three functions: (ⅰ) PUFA ω-3 is beneficial for heart health [36]; (ⅱ) PUFA ω-3 can regulate blood levels and help maintain healthy blood lipid levels [37]; (ⅲ) PUFA ω-3 can improve brain function [38]. PUFA ω-3 has been shown to protect BBB integrity and inhibit glial and inflammatory activation in a mouse model of Alzheimer’s disease. In addition, PUFA ω-3 can penetrate the BBB [39, 40]. In the present study, dietary PUFA ω-3 supplementation reduced sodium nitrite-induced fetal BBB, brain tissue, and mitochondrial damage. Reportedly, PUFA ω-3 supplementation during pregnancy may benefit fetal brain development. No clinically relevant side effects were reported [41]. However, the use of drugs should be moderate and targeted. Excessive PUFA ω-3 may cause low blood sugar levels and intestinal discomfort. In addition, some individuals may be allergic to PUFA ω-3. According to an individual’s constitution, it is crucial to supplement PUFA ω-3 appropriately and in a targeted manner. In future studies, we plan to further investigate the effects of different PUFA ω-3 doses on fetal rat brain development.

In late-onset preeclampsia, maternal PUFA ω-3 and vitamin E supplementation can improve placental angiogenesis [42]. Meanwhile, in pregnant women lacking unsaturated fatty acids, PUFA ω-3 supplementation during pregnancy could benefit the prognosis [43]. In the study, the ratio of live fetuses, average body weight, and brain weight increased after PUFA ω-3 treatment, suggesting that PUFA ω-3 was effective against changes induced by excessive sodium nitrite exposure during pregnancy.

In preeclampsia and gestational marginal zinc deficiency studies, pregnant rats were sacrificed at E19 [44, 45]. In the present study, pregnant rats were treated from E0 to E19, but we did not study the effects of drugs on fetal rats post E19. Additional studies are needed to examine the effects of sodium nitrite and PUFA ω-3 treatment on fetal rats after E19.

## 5. Conclusions

PUFA ω-3 supplementation might alleviate placental and cerebral cortex tissue inflammation, adverse pregnancy outcomes, impairment of BBB function, neurological impairment of the brain, oxidative stress damage, and mitochondrial damage caused by excessive exposure to sodium nitrite during pregnancy. These effects might be related to the NRF1/HMOX1 pathway. The present study provides evidence from rat models indicating that PUFA ω-3 supplementation during pregnancy could support the health and development of the fetus.

## Supporting information

S1 Fig Experimental design of protocols 1 and 2. (TIF)

S2 Fig The uncropped original blot of (A) Fig 4C and (B) Fig 4F. Each lane was labeled according to the cropped blots in Fig 4C and 4G.
(TIF)

S1 Dataset All data generated during this study. (XLSX)

S1 Raw images (PDF)

## Abbreviations

ATP: Adenosine triphosphate
ALA: alpha-linolenic acid
BBB: Blood-brain barrier
mt-Co1: *cytochrome c oxidase I*, *mitochondrial*
DHA: Docosahexaenoic acid
EPA: Eicosapentaenoic acid
E: Embryonic day
ENO2: enolase 2
ELISA: Enzyme-linked immunosorbent assay
H&E: Hematoxylin & eosin
HMOX1: Heme oxygnase 1
Hmox1: *Heme oxygnase 1*
IF: Immunofluorescence
IHC: Immunohistochemistry
NOS2: nitric oxide synthase 2
Nos2: *nitric oxide synthase 2*
IOD: Integrated optical density
IL: 1B interleukin-1beta
Il1b: *interleukin 1 beta*
MDA: Malondialdehyde
NO: Nitric oxide
NRF1: Nuclear respiratory factor 1
Nrf1: *Nuclear respiratory factor 1*
OCLN: Occludin
PUFA: ω-3 Omega-3 polyunsaturated fatty acids
ANOVA: One-way analysis of variance
OD: Optical density
ONOO: Peroxynitrite anion
qRT-PCR: Quantitative real-time PCR
ROS: Reactive oxygen species
rpm: revolutions per minute
SOX2: Sex determining region Y-box 2
SDS-PAGE: Sodium dodecyl sulfate-polyacrylamide gel electrophoresis
SDSprague: Dawley
Sdha: *Succinate dehydrogenase complex flavoprotein subunit A*
SOD: Superoxide dismutase
JC-1: Tetraethyl benzimidazolo carbocyanine iodide
TJP1: tight junction protein 1
TEM: Transmission electron microscope
TNF: Tumour necrosis factor
Tnf: *Tumour necrosis factor*
VDAC: Voltage-dependent anion channel
