# Supplementary figures and images for: Supplementation with dietary omega-3 PUFA mitigates fetal brain inflammation and mitochondrial damage caused by high doses of sodium nitrite in maternal rats
Source: PLoS One. 2022 Mar 24;17(3):e0266084. doi: 10.1371/journal.pone.0266084 (PMC8947126; doi:10.1371/journal.pone.0266084)

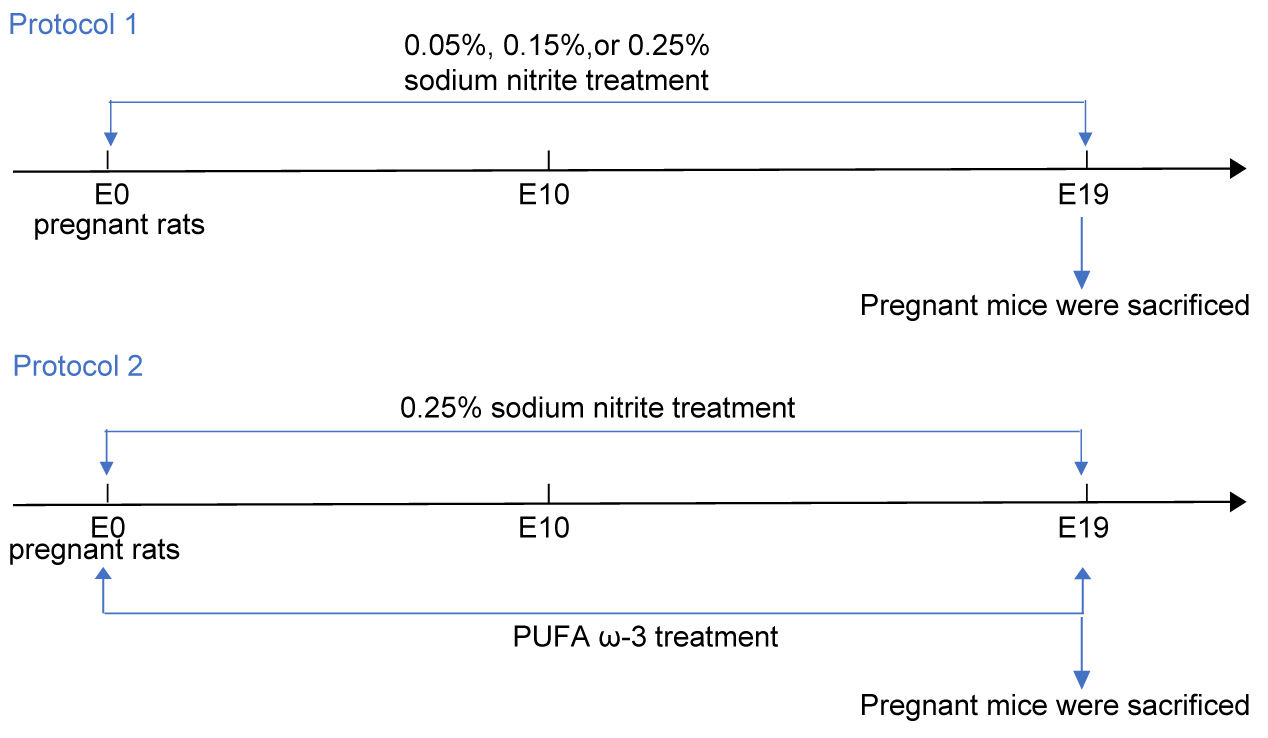

Supplement: S1 Fig — (TIF) [file pone.0266084.s001.tif]

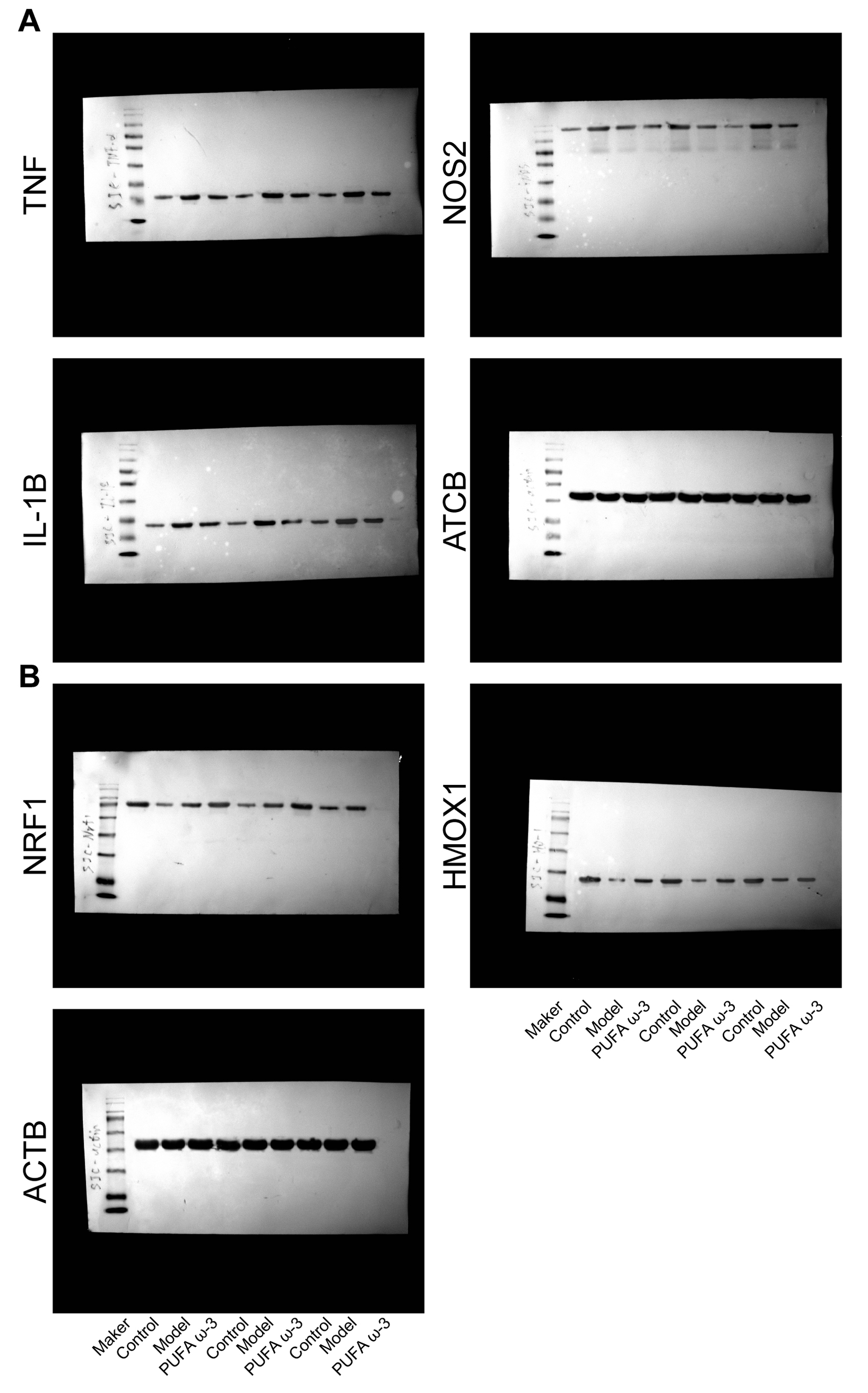

Supplement: S2 Fig — Each lane was labeled according to the cropped blots in Fig 4C and 4G. (TIF) [file pone.0266084.s002.tif]

**Figure 3A**

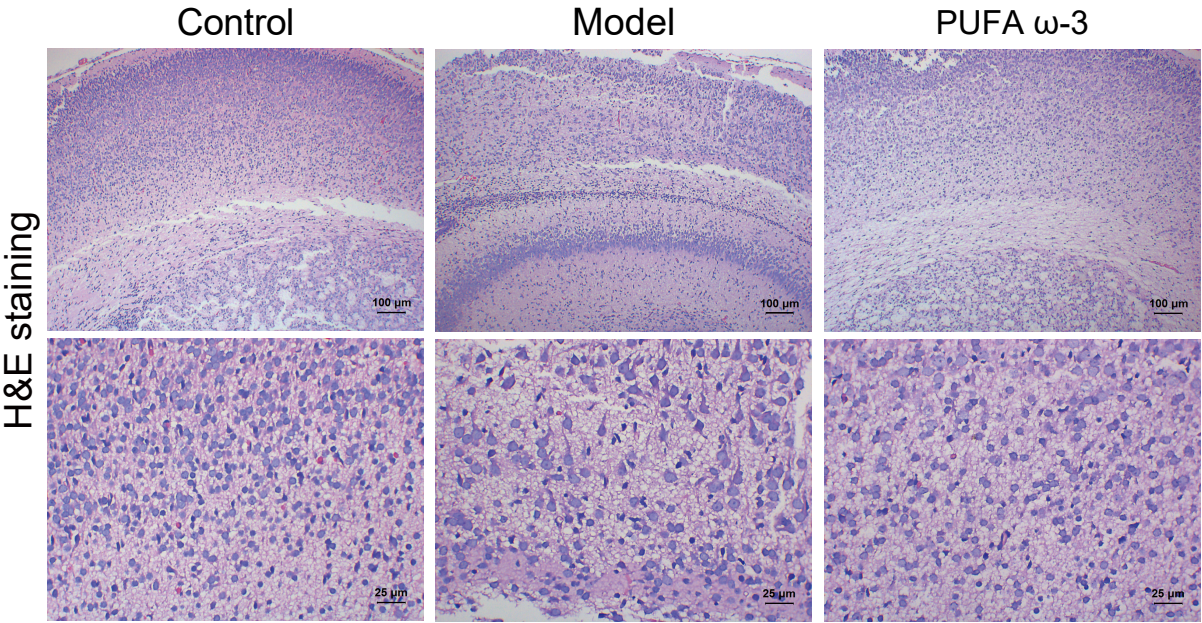

**Figure 3B**

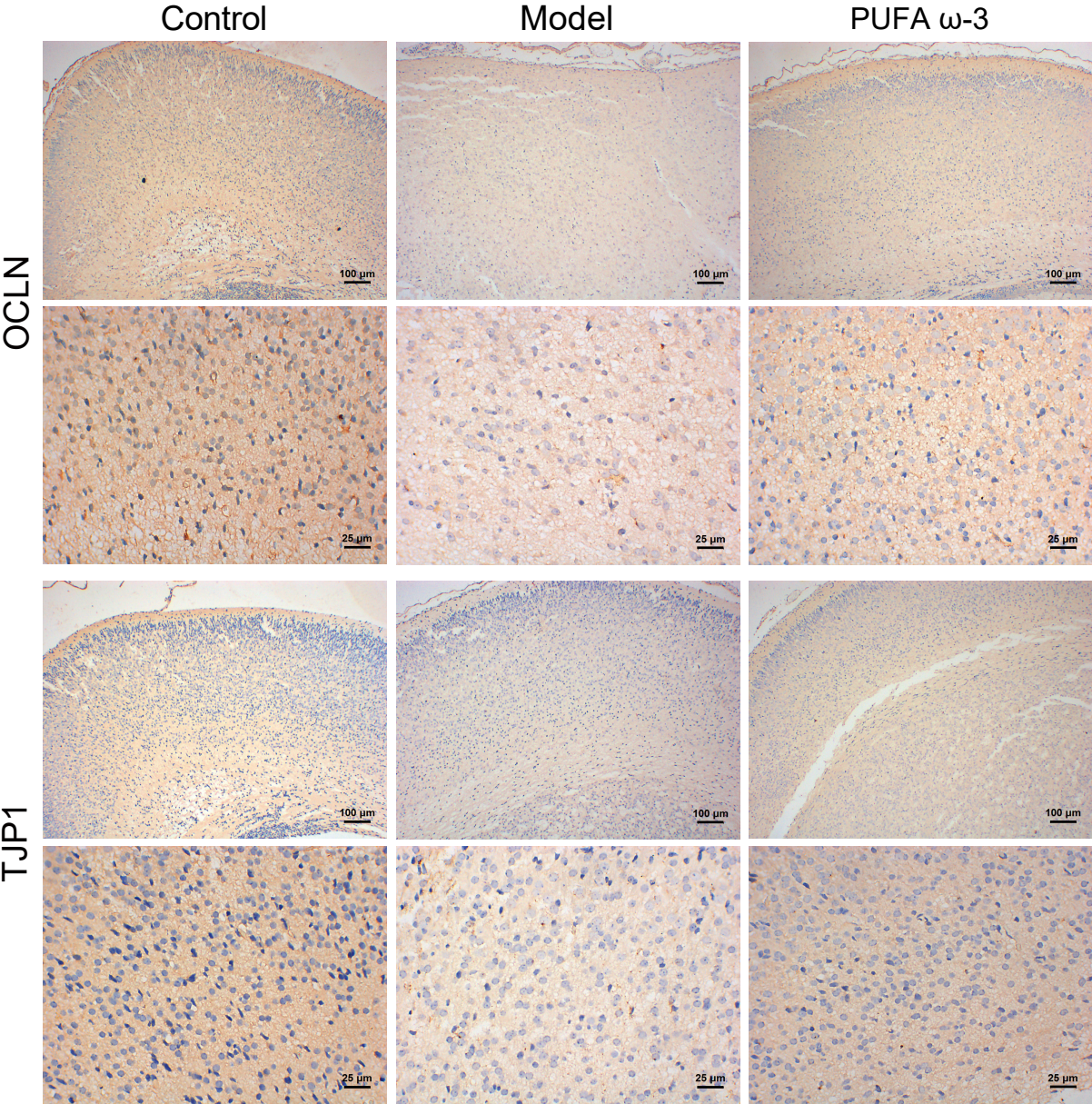

Figure 4D

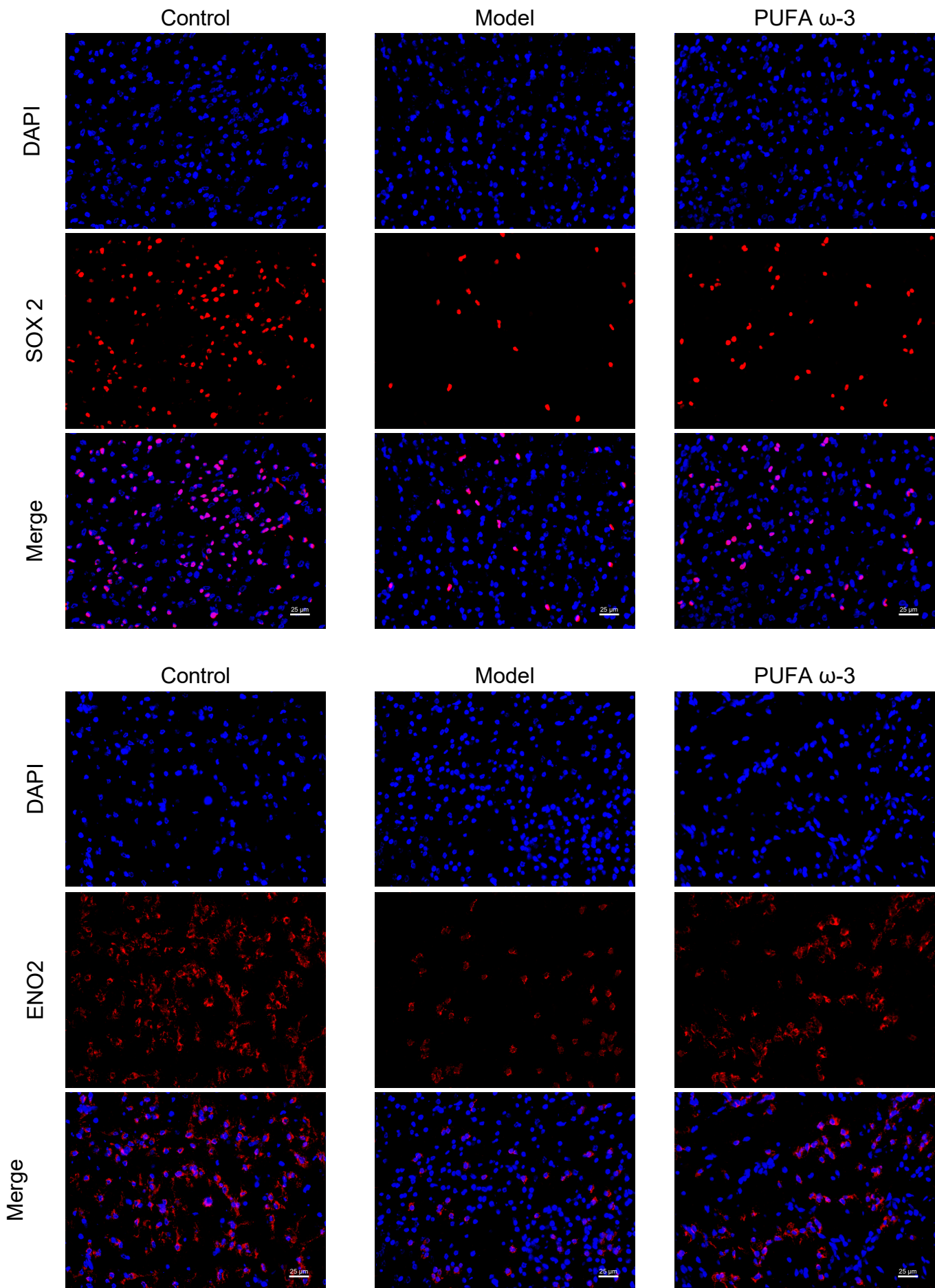

**Figure 4D**

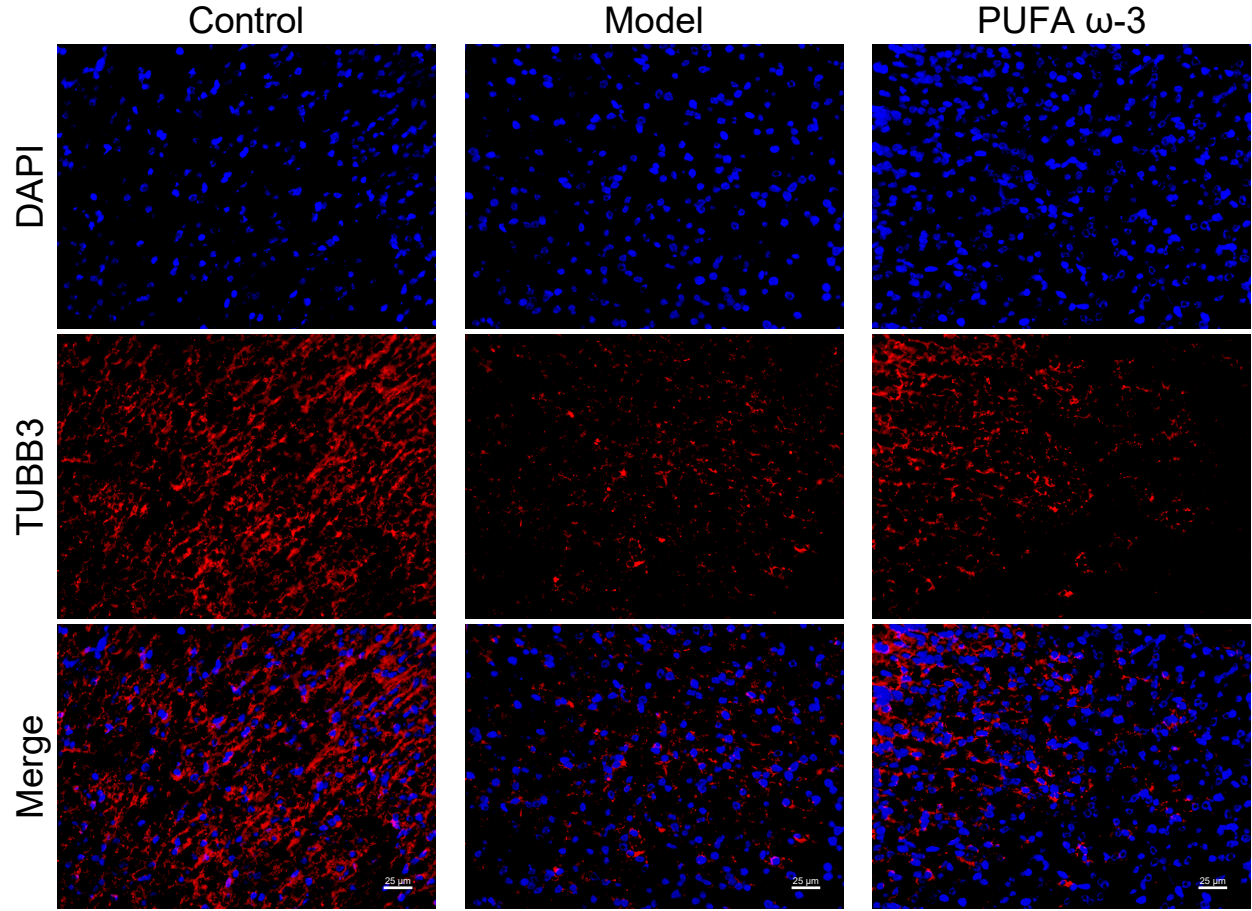

**Figure 5G**

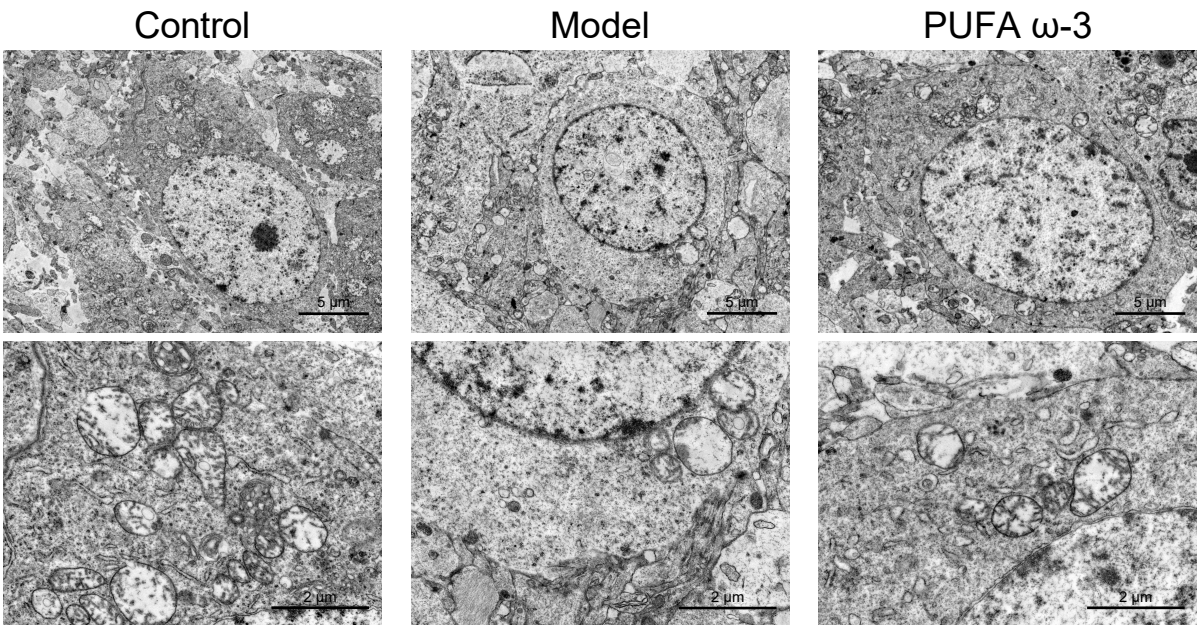

Supplement: S1 Raw images — (PDF) [file pone.0266084.s004.pdf]
